# Report on Practical Medicine

**Published:** 1870-09

**Authors:** James S. Whitmire

**Affiliations:** Metamora, Ill., one of the Committee


### ARTICLE XXV.

### REPORT ON PRACTICAL MEDICINE.

By JAMES S. WHITMIRE, M.D., of Metamora, Ill., one of the Committee.

Mr. President and Gentlemen of the Illinois State Medical Society:

Not having been apprized by your Chairman of this Committee, of any specified plan that he wished to have carried out in this report, I have been compelled, in this, to draw from my own resources, and those of the physicians living in Mason, Tazewell, Woodford, Marshall, and Livingston Counties. I, therefore, as one of your Committee beg leave to submit the following report:—

The Counties above named, all, with the exception of Livingston, border upon the Illinois River, its eastern shore being their western boundary; hence, in the variety and character of their soil, they are not surpassed by any equal number of Counties in the State.

The topography of these Counties is the most varied and beautiful; they being interspersed with natural groves, with, here and there, the most enchanting streams and rivulets, that wend their way to the placid waters of the quiet Illinois. The praries are gently undulating, and are now subdued by the magic hand of civilization, till they do, in fact, blossom as the rose and yield their rich fruits in harvest to the frugal husbandman.

Besides the Illinois River on the west, these counties have, traversing their full extent, the Sangamon, Mackinack, and Vermillion Rivers, and the lesser streams of Salt Creek, Sugar and Crow Creek, to say nothing of an immense number of still smaller streams, that are known only in their separate localities. More than two-thirds of this vast extent of country is prairie, which is well settled and under thorough cultivation. Its altitude above the city of Cairo, at the confluence of the Ohio and Mississippi Rivers, is about 600 feet. The water mostly used by the inhabitants, for culinary and other purposes, is limestone, or "hard water," as it is usually called. The lands of these counties are generally arable and produce the finest crops of the different cereals.

There is but a small quantity of bog, or marshy lands, in these counties, and they are situated along the Illinois River, most of which are subject to the annual overflow of that stream, rendering them useless for any purpose, except that of stock range, the latter part of the season.

During the year 1869, there was more rain fell in this section than probably ever fell before, in the same time; and had the season been hot, as well as wet, we might naturally have expected a great amount of sickness; but as it was, there has never passed as healthy a season, since I have lived in the district, which is now 24 years past. During this rainy season, which lasted till near the last of July, there was only the ordinary sickness of the spring months, such as rheumatism, bronchitis, croup, etc., with an occasional attack of intermittent fever; and these easily yielded to the usual remedies, there being nothing particular or striking in either their character or type.

During the months of August and September, however, the mercury ranged from 82° to 96° Fahrenheit, at noon; so that the whole surface of the earth which, till now, seemed a common cesspool, suddenly dried up, so that there was no chance, in most localities, for the production of malarial poison; hence, during this time, we had but little more than the usual amount of malarial fevers. Many of the cases, however, that did occur, were of the typho-malarial type, and, consequently, yielded less readily to treatment, than the simple remittent fevers of former years.

The winter of 1869–70 substantially set in about the 1st of November, and bid fair, at the start, to be an unusually severe one. It proved, however, to be a long one, but the mildest for many years; the frost being out of the ground by March 20th, 1870; mercury never having dropped, during the whole time, below 6°, below 0. During this time, we had an unusual quantity of acute and typhoid pneumonia, acute and sub-acute bronchitis, croup, rheumatism, etc., none of which exhibited any special peculiarities; unless, perhaps, there was exhibited a greater amount of malarial influence connected with them, than has been common of late years, in our ordinary winter fevers, and the grade of vitality of our patients was possibly lower than usual. These conditions, however, being met by their appropriate remedies, we found the diseases of last winter, notwithstanding their prevalence, equally as amenable to remedies, as on other occasions, and the mortality was no greater than at other times, or seasons. Our treatment of these cases of lowered vitality, as well as those presenting malarial influences, has not differed from that usually pursued by the profession; hence, in a supplemental report like this, we think it would add nothing to the interest of the Society, to give it in detail.

### INFECTIONS AND EPIDEMICS.

The village and vicinity in which I reside—Metamora, the county seat of Woodford county—has had no visitations of this character of diseases for several years. During the past winter, however, the eastern portion of this county, and LaSalle, Livingston, Tazewell, and Marshall counties have all been pretty thoroughly scourged with scarlatina; it appearing in those counties, in certain neighborhoods, as an epidemic, and presenting itself in its most malignant form. This was the case more particularly in the eastern portion of Woodford, southern

LaSalle, and northern Livingston. The facts, in regard to this scourge, I have learned through the favors of Drs. Wilcox and Stoner, of Minonk, and Drs. Lewis and Cole, of El Paso, all of whom have been extensively engaged in the treatment of the malady. They inform me that, so far as they can see, this epidemic has not differed, in any particular, from others that has formerly appeared, nor has it been either more or less fatal than in its previous visitations. Their treatment has been of a sustaining character, such as iron, quinine, chlorate of potassa, etc., internally, with mild laxatives, when necessary, of castor oil, rhubarb, etc.; and, in case the bowels are too lax, which, in this epidemic has been frequently the case, they have derived great advantage from the terebinthinate emulsion, in connection with creosote and laudanum. Their patients were sponged every day with warm alkaline water, and kept in a clean, airy room, with plenty of beef-tea or milk as food. Under this regimen and treatment, their patients generally recovered. I will simply remark, in this place, that I have used a gargle or throat-wash, in this and similar affections, that, in my opinion, is a great improvement over anything that I have seen recommended for this purpose. It is made as follows:—

| | | |
|---|---|---|
| ℞. | Alcohol, | ℥ss. |
| | Elix. of Vitriol, | ʒij. |
| | Sulp. Alum, | ʒj. |
| | Creosote, | gtt., XL. |
| | Ol. Cinnam., | gtt., xxx. |
| | Syrup Simp., | ℥iijss. |

The patient, if strong, or old enough, may gargle with this mixture, or the fauces and pharynx may be swabbed by an assistant; and though this will be found to be pretty sharp at first, yet the comfort that immediately follows its use, makes the patient not only reconciled, but ask to have it used.

Aside from the prevalence of this epidemic, in the above counties, there has been no prevalence of any other than their ordinary winter fevers; and they have shown much the same characteristics as the diseases in our vicinity.

During the latter part of March, we have had in our practice two well-marked cases of cerebro-spinal meningitis, both children, one 12, a boy, the other a girl 14 years of age. The nature, course, and character of this dreadful disease are now so well understood by the profession, that it would be useless, in this place, to occupy the time of this Society with their details. But I would beg the indulgence of the Society, while I give, in as succinct a manner as possible, the principle of my treatment of these two cases. The treatment of this disease usually resorted to by the profession has, in my hands, and in those too of my compeers, proven so unsatisfactory, that I deemed it no disrespect to authority, to step out of the usual precincts of ordinary government, in such cases, and explore new fields for the benefit of myself, of science, and, more particularly, for the benefit of my patients. I, therefore, determined, with a full conviction of success constantly before me, to try what virtue there was in the use of hydrate of chloral, in quieting the restlessness that attends this disease. I wished also to produce sleep, allay pain along the spine, and relieve the apparent stricture around the chest, to moderate the frequency of the heart's action, and to relieve the permanent opisthotonos, that is one of the ever present characteristics of this disease. Certainly, this is one of the most formidable diseases with which the profession has to deal, and, with the most approved treatment, there is still a large per cent. of mortality to be recorded; hence, I felt perfectly justified in using any remedy, however lately it may have been brought to the notice of the profession, if, in my judgment, its acknowledged therapeutic action seemed to point towards the fulfilment of the indications to be met in this disease. Here, before me, was one of the most dangerous diseases that flesh is heir to. In my judgment, I saw in the hydrate of chloral one of our most formidable remedies, just suited to fill all the indications of treatment, necessary to give nature a chance to rally and overcome the diseased condition. And I am now pleased to be able to state to the profession, that from the favorable action of this remedy in these two cases, I consider this drug, in the treatment of this disease, a great acquisition to our stock of heroic remedies.

After thorough mercurial purgation, assisted by saline cathartics, I gave to these children from 10 to 15 grains of chloral, and one drachm of the officinal syrup of ipecacuanha, dissolved in 2 ounces of water, every three, four, or five hours, as the exigencies of the cases seemed to require. I enveloped the patients in a warm, wet sheet for an hour, once or twice per day, and laid around my patients, at the time, hot boiled ears of corn. When they were not in the pack, I kept warm, emolient poultices constantly applied to the spine. The bowels were kept in a soluble condition, when necessary, by the use of Sal. Rochelle, which, by the way, I will here remark, is seldom necessary, on account of the hyd. of chloral having a tendency itself to relax the bowels.

In the course of from three (3) to five (5) days, the most urgent symptoms began to subside, the pulse became regular, soft, and natural, the contraction of the dorsal muscles became relaxed, the skin became soft and natural, the urine, which had been scanty, was augmented in quantity, and the appetite began to return, so that in the course of seven or eight days from the commencement of the treatmemt, convalescence was fully established, and my patients, as I am fully satisfied, snatched from impending death. After the most urgent symptoms were allayed, say about the sixth day, I gave my patients one grain of quinine, three times per day, with a little Dover's powder; and at the usual bedtime, I ordered a dose of chloral, in order to insure tranquility and sleep, without which there can be no recuperation. This gentlemen, it is true, is a history of but two cases of cerebro-spinal meningitis, treated with this new and powerful remedy. The success was complete. I give them for what they are worth, and hope thereby to induce the profession to give this drug, in the treatment of this disease, a fair and impartial trial; and if, upon investigation by the light of experience, we have lessened the per cent. of mortality, in this disease, we will have disarmed it of more than half its terrors, and imbued the profession with a confidence hitherto unknown in the treatment of this terrible malady.

Just now, my attention has been called, while writing this article, to a case of chorea, in a girl of 14 years of age, whom my brother, Z. H. Whitmire, has been treating for some two weeks. She is a delicately organized creature, and naturally very easily excited.

She has been taking chalybeates, nervines, laxatives, etc., without the least benefit whatever, and the young lady is manifestly growing worse from day to day, so that the family are in constant terror lest she may go into convulsions at any moment. I recommended hyd. chloral, gr. x, in aqua, ℥ij, at bedtime, each night; the first dose of which produced the first tranquil sleep the poor sufferer has had for more than a week. She is continued on Griffith's myrrh mixture, with assafœtida and quinine, during the daytime, and takes the chloral at night, which has constantly, as in the first instance, produced quiet sleep. She is becoming much more calm during the day, and is, at this writing, apparently improving. Certainly, in this case, whether the child is permanently benefited or not, this drug has acted like magic in quieting the unruly muscles, and in allaying the alarm of the friends.

*May 22.* This patient is completely restored to health at this date.

